# The Biochemistry of Sensing: Enteric Pathogens Regulate Type III Secretion in Response to Environmental and Host Cues

**DOI:** 10.1128/mBio.02122-17

**Published:** 2018-01-16

**Authors:** Nicole J. De Nisco, Giomar Rivera-Cancel, Kim Orth

**Affiliations:** a Department of Molecular Biology, University of Texas Southwestern Medical School, Dallas, Texas, USA; b Howard Hughes Medical Institute, University of Texas Southwestern Medical School, Dallas, Texas, USA; c Department of Biochemistry, University of Texas Southwestern Medical School, Dallas, Texas, USA; National Cancer Institute

**Keywords:** T3SS, cell signaling, enteric pathogens, environmental cues, nutritional stress, pathogenesis, surface sensing

## Abstract

Enteric pathogens employ sophisticated strategies to colonize and infect mammalian hosts. Gram-negative bacteria, such as *Escherichia coli*, *Salmonella*, and *Campylobacter jejuni*, are among the leading causes of gastrointestinal tract infections worldwide. The virulence strategies of many of these Gram-negative pathogens rely on type III secretion systems (T3SSs), which are macromolecular syringes that translocate bacterial effector proteins directly into the host cytosol. However, synthesis of T3SS proteins comes at a cost to the bacterium in terms of growth rate and fitness, both in the environment and within the host. Therefore, expression of the T3SS must be tightly regulated to occur at the appropriate time and place during infection. Enteric pathogens have thus evolved regulatory mechanisms to control expression of their T3SSs in response to specific environmental and host cues. These regulatory cascades integrate multiple physical and chemical signals through complex transcriptional networks. Although the power of bacterial genetics has allowed elucidation of many of these networks, the biochemical interactions between signal and sensor that initiate the signaling cascade are often poorly understood. Here, we review the physical and chemical signals that Gram-negative enteric pathogens use to regulate T3SS expression during infection. We highlight the recent structural and functional studies that have elucidated the biochemical properties governing both the interaction between sensor and signal and the mechanisms of signal transduction from sensor to downstream transcriptional networks.

## INTRODUCTION

Gastrointestinal (GI) tract infections caused by enteric pathogens affect over 1.7 billion individuals annually, with approximately 2.2 million cases ending in death (1). Severe diarrheal disease resulting from GI tract infections is a leading cause of death for children under 5 years (2). Many developed countries have significantly reduced their incidence of foodborne outbreaks, but factors including globalization, environmental change, and increasing antibiotic resistance are facilitating the rapid reemergence and spread of severe enteric pathogens (3). The causal agents of gastrointestinal tract infections include bacteria, viruses, and eukaryotic parasites (1, 2). Most GI tract infections, even those caused by bacteria, cannot be treated effectively by current antibiotic therapies because the antibiotics are either ineffective, cause severe dysbiosis of the intestinal microbiota, or trigger serious complications, such as septicemia from antibiotic-induced endotoxin release (3). Thus, new strategies must be employed to develop antimicrobial therapies that will be effective and safe in the treatment of infections caused by enteric bacterial pathogens (4, 5).

Enteric bacterial pathogens span several genera, including *Escherichia*, *Salmonella*, *Shigella*, *Yersinia*, *Vibrio*, and *Campylobacter*. These species occupy different environmental habitats and use diverse domestic and wild animals as reservoirs (6). The oral sources of infection by these pathogenic bacteria vary as widely as their environmental niches. *Salmonella* and *Campylobacter* infections are usually acquired from undercooked chicken and eggs, while GI tract infections caused by *Yersinia* species are usually acquired from undercooked pork and vegetables (3, 6–8). Many *Vibrio parahaemolyticus* infections are caused by contaminated raw or undercooked seafood, and *Vibrio cholerae* infections are typically caused by contaminated drinking water (9, 10). Enteropathogenic and enterohemorrhagic *Escherichia coli* (EPEC and EHEC, respectively) infections, as well as *Shigella* infections, are linked to consumption of contaminated food and water (11). Interestingly, these pathogens colonize different parts of the GI tract. For example, *V. cholerae* and EPEC prefer the small intestine, whereas *Campylobacter jejuni* and EHEC infect the cecum and colon, respectively (12, 13).

Toxin delivery systems are key to the environmental survival and persistence of enteric pathogens, as well as to their mechanisms of pathogenesis within human hosts (14). An important toxin delivery system used by many Gram-negative pathogens is the type III secretion system (T3SS). The T3SS is a macromolecular injectisome that translocates bacterial toxins, termed effectors, directly from the bacterial cytoplasm into the host cytosol (15). The expression of the T3SS apparatus and effector proteins must be tightly regulated to coincide with both the appropriate host cell environment and correct stage and location of infection (16). To achieve this coordination, bacteria have evolved mechanisms to sense different physical and chemical host signals and integrate the sensing of these signals into the regulatory control of pathogens via the T3SS apparatus and effector expression. This review focuses on the signals and sensors mediating T3SS expression during gastrointestinal tract infections.

## TEMPERATURE

**FIG 1:**
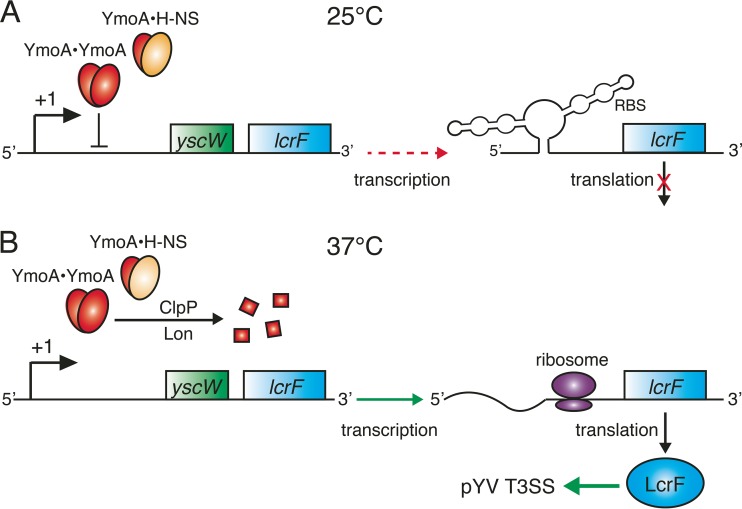
Thermosensing by intergenic RNAT and thermolabile protein YmoA in *Y. pseudotuberculosis*. (A) At moderate temperatures (25°C), the transcription of the *yscW-lcrF* operon is partially repressed by YmoA homodimers and/or YmoA–H-NS heterodimers. Translation is fully repressed by a two-stem-loop structure in the 5′-UTR of the *lcrF* mRNA, the RNAT. (B) At 37°C, the thermolabile protein YmoA is rapidly degraded by ClpP and Lon proteases, and this lifts the transcriptional repression of *lcrF*. High temperatures also melt the inhibitory two-stem-loop structure in the *lcrF* mRNA, allowing for enhanced translation of LcrF, the transcriptional activator of the pYV-encoded T3SS genes.

For bacteria to survive and thrive in diverse environments, they must be able to sense and respond to changes in temperature (17). For intestinal pathogens, temperature change is a primary indicator for successful invasion of a mammalian host (18). Therefore, temperature-sensing mechanisms are almost ubiquitously integrated into the regulatory circuits governing virulence gene expression of human intestinal pathogens (18). Many temperature-sensing mechanisms exist in bacteria. These include temperature-induced structural changes in intrinsically bent or supercoiled DNA, thermosensing regulatory proteins, and RNA thermometers (RNATs) (18–20). RNATs are RNA sequences present in the 5′-untranslated region (UTR) of certain mRNAs that adopt ribosomal binding site-occluding secondary structures at restrictive temperatures but not at permissive temperatures (21). The intestinal pathogens *Yersinia pseudotuberculosis* and *Yersinia enterocolitica* utilize these temperature-sensing strategies to efficiently regulate the expression of virulence factors, including the T3SS, within mammalian hosts (22). In pathogenic *Yersinia* species, the T3SS genes are encoded on the pYV virulence plasmid. Like the majority of the pYV-encoded virulence genes, the T3SS genes are not expressed at temperatures below 30°C and are most highly expressed at 37°C (23). The expression of the T3SS-encoding *ysc* genes (*yscA* to *yscL*), as well as of the *yop* effector genes (*yopE*, *yopH*, *yopK*, and *yopO*), is activated by the AraC-type regulator LcrF, which responds to both temperature and host cell contact (24–26). The *lcrF* gene is in an operon with *yscW*, and their transcription is controlled by a promoter sequence upstream of *yscW* (Fig. 1A).

In *Y. pseudotuberculosis*, temperature regulation of the *ysc*/*yop* regulon is achieved by the combined action of temperature-dependent proteolysis of the transcriptional repressor YmoA and an RNAT within the *lcrF* 5′-UTR (20). At moderate temperatures, similar to what a bacterium might experience outside a mammalian host (25°C), expression of the *yscW*-*lcrF* operon is repressed by two distinct mechanisms (Fig. 1A). First, the transcriptional repressor YmoA, which can form a homodimer or a heterodimer with the nucleoid-associated protein H-NS, binds to sequences downstream of the transcription initiation site and partially represses transcription of the *yscW*-*lcrF* operon (Fig. 1A) (20, 27). Second, any transcribed *lcrF* mRNA is not translated because of a two-stem-loop structure that forms in the 5′-UTR at moderate temperatures. This RNA structure masks the Shine-Dalgarno sequence, thereby preventing the binding of the 30S ribosomal subunit, ribosome assembly, and translation of the *lcrF* mRNA (Fig. 1A) (20). Without the transcriptional activator LcrF, the expression of the genes encoding the T3SS apparatus and effectors is not induced (Fig. 1A) (23, 28). At higher temperatures, such as those that would be encountered within mammalian hosts (37°C), both types of repression of *lcrF* expression are relieved. Transcriptional repression of the *yscW-lcrF* operon is lifted by the rapid, temperature-dependent degradation of YmoA by both ClpXP and Lon proteases at 37°C (Fig. 1B). Several mechanisms for the temperature-dependent degradation of YmoA have been proposed. These include increased expression or activity of ClpXP or Lon proteases at 37°C, conformational changes in YmoA that make it a better substrate for ClpXP or Lon at higher temperatures, or direct modification and targeting of YmoA for degradation by an accessory protein at 37°C. Another possible mechanism is that Lon and ClpXP have differing affinities for YmoA homodimers and YmoA–H-NS heterodimers, and the higher-affinity species may dominate at elevated temperatures (29). Translational repression of the *lcrF* mRNA is also relieved at 37°C, because the inhibitory two-stem-loop structure melts at this temperature, thereby revealing the sequestered Shine-Dalgarno sequence and allowing translation of LcrF (Fig. 1B) (20). LcrF then induces production of the T3SS by binding to specific TTTaGYcTtTat (highly conserved nucleotides are in capital letters) DNA motifs in the promoter regions of many T3SS genes of the *ysc/yop* regulon and activating their expression (Fig. 1B) (30, 31).

## HORMONES

The locus for enterocyte effacement (LEE)-encoded T3SS of EHEC, which is essential for formation of the characteristic attaching and effacing (AE) lesions, is regulated by two host hormones, epinephrine (Epi) and norepinephrine (NE), and by an autoinducer (AI-3) that is produced by the gut microbiota. Epi and NE are at the core of stress response signaling in humans and are recognized by G-protein-coupled receptors in mammalian cells (32). However, bacteria lack homologues of these mammalian adrenergic receptors and have instead evolved sensor histidine kinases (HKs) to recognize these host signals. These chemical signals are received and transmitted by two HKs, QseC and QseE (33–35). Why EHEC and other enteric pathogens have developed mechanisms to sense Epi and NE remains unclear, but given the important functions neurotransmitters play in gut homeostasis, it is thought that sensing these molecules may allow the pathogen to assess the fitness of the host and modulate its virulence program accordingly (36). In fact, it has been shown that a *qseC qseE* double mutant is attenuated in an EHEC infant rabbit infection model (36). The importance of *qseC* and *qseE* in virulence has also been demonstrated in *Citrobacter rodentium*, a murine pathogen that harbors a LEE-encoded T3SS (36). *C. rodentium* LEE expression and gut colonization are both diminished in *Dbh*^*−/−*^ mice, because they do not produce Epi and NE (36).

**FIG 2:**
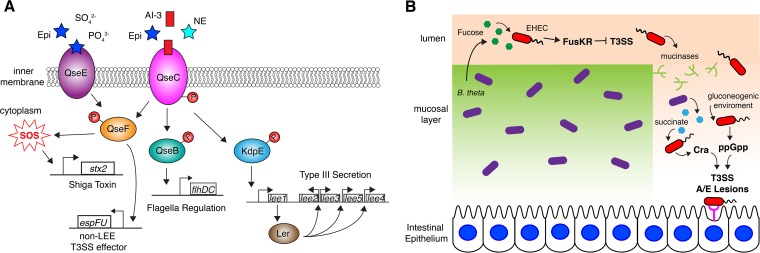
Regulation of the LEE-encoded T3SS by hormone and nutritional sensing in EHEC. (A) The histidine sensor kinase QseC binds host hormones Epi and NE as well as the microbiome-produced AI-3. Upon ligand binding, QseC autophosphorylates and transfers phosphate to the RRs QseB, QseF, and KdpE. P-KdpE activates expression of the LEE-encoded T3SS through Ler. P-QseB modulates flagellar operons, while P-QseF induces expression of the non-LEE T3SS effector EspFU and activates the SOS response, which in turn activates *stx2* expression. QseF is also phosphorylated by the sensor HK QseE in response to Epi, SO_4_^2−^, and PO_4_^3−^. (B) Within the intestinal lumen, EHEC senses fucose produced by *B. thetaiotaomicron* through the HK/RR pair FusKR, which represses T3SS expression when fucose is present. To reach the intestinal epithelium, EHEC produces mucinases that obliterate the mucosal layer and create a gluconeogenic environment. *B. thetaiotaomicron* then switches to gluconeogenic metabolism and secretes large amounts of succinate, which EHEC senses through the transcriptional regulator Cra. Sensing of succinate by Cra induces T3SS expression and AE lesion formation. The gluconeogenic environment also triggers EHEC’s stringent response, during which the alarmone ppGpp is synthesized. ppGpp directly binds RNA polymerase and modulates its activity, such that LEE expression is upregulated.

For EHEC, AI-3, Epi, and NE are first sensed by the membrane-bound HK QseC, resulting in autophosphorylation and transfer of a phosphate to its cognate response regulator (RR), QseB. Phosphorylated QseB (P-QseB) then activates the transcription of the *qseB-qseC* operon as well as the flagellar motility operon *flhD-flhC* (Fig. 2A) (32, 34). QseC can also phosphorylate the noncognate RRs QseF and KdpE (32, 37). P-KdpE directly activates transcription of *ler*, which encodes the master transcriptional activator of all LEE genes (37). QseC autophosphorylation in response to AI-3, Epi, or NE also activates the expression of the sensor HK QseE, which can itself sense Epi, sulfate, and phosphate (Fig. 2A). Both QseC and QseE can phosphorylate the RR QseF, thereby regulating the expression of the non-LEE-encoded effector T3SS EspFu, which is directly involved in AE lesion formation. QseC also indirectly induces expression of the λ phage-encoded Shiga toxin (*stxAB*) through the QseF RR by activating the SOS response. During the SOS response, expression of *recA* is upregulated and, through its coprotease activity, RecA cleaves the λ cl repressor of the *stxAB* genes, allowing for their expression (Fig. 2A) (32, 38).

The structural basis by which QseC or QseE senses AI-3, Epi, or NE remains undefined. Although the crystal structure of the cytoplasmic domain of QseC is available, it does not include the periplasmic sensor region. Recently, Parker et al. used software to generate a predicted structure of the periplasmic domain of QseC (39). The authors identified 8 conserved residues in the periplasmic domain that may mediate ligand binding. These residues reside within a potential binding pocket formed by a series of β-sheets and proximal α-helices. However, mutation of these residues did not abrogate the ability of QseC to sense Epi and NE (39). Future structural studies of QseC and its ligands, NE and Epi, are required to completely understand how these signals are sensed.

## SUGARS AND NUTRITIONAL STRESS

While inside the host gastrointestinal tract, enteric pathogens must not only adapt to the nutritional conditions of the gut but also use these nutritional cues to direct the appropriate expression of virulence factors. Pathogenic bacteria that colonize the colon (e.g., EHEC) do not have access to simple dietary sugars because these sugars are absorbed in the small intestine (40). These pathogens must compete with a specialized microbiota that resides in the lumen and outer mucous layer of the intestine. Many members of the microbiota can use the abundant undigested plant polysaccharides and host glycans found in the colon (40). However, neither commensal nor pathogenic *E. coli*, such as EHEC, can use polysaccharides. EHEC avoids competition with commensal *E. coli* for monosaccharides and disaccharides by invading the mucosal layer and adhering to enterocytes of the intestinal epithelium (41). To achieve this, EHEC must sense and appropriately respond to a variety of environmental signals.

A prominent member of the luminal microbiota, *Bacteroides thetaiotaomicron*, produces multiple fucosidases that cleave fucose from the host glycans comprising the mucosal layer (42). Initially, EHEC senses the high concentration of fucose through the HK and RR pair FusKR (Fig. 2B). Activation of the FusKR signaling cascade by fucose represses transcription of several genes, including Ler, the master regulator of the LEE-encoded T3SS (43). The downregulation of the T3SS and other LEE-encoded virulence factors, which are unnecessary for EHEC’s survival in the lumen, is a crucial energy-saving measure that helps EHEC outcompete commensal *E. coli*. During the second stage of its virulence strategy, EHEC senses other *B. thetaiotaomicron* metabolites (e.g., succinate) and ramps up expression of mucinase, which clears the mucous layer of the intestine and opens a path to the intestinal epithelium (Fig. 2B). Without the mucous layer as a primary carbon source, *B. thetaiotaomicron* switches to a gluconeogenic metabolism and secretes large amounts of succinate. The high levels of succinate are sensed by EHEC via an unknown mechanism and induce the transcriptional regulator Cra, which in turn activates expression of the T3SS (44).

The gluconeogenic, nutrient-poor environment also stimulates EHEC’s stringent response (Fig. 2B). The stringent response is a universal bacterial transcriptional regulatory mechanism that involves synthesis of the alarmone ppGpp by the RelA and SpoT enzymes during times of nutrient deprivation (45). ppGpp, along with the starvation-induced small protein DksA, directly binds to and modulates the activity of RNA polymerase. This activates or represses transcription at specific promoters, resulting in upregulation of LEE expression in EHEC (Fig. 2B) (46). The double activation of LEE expression by both Cra and ppGpp in response to the nutrient-poor conditions at the intestinal epithelium ensures that the LEE-encoded T3SS is expressed at the appropriate stage of infection.

## CATIONIC ANTIMICROBIAL PEPTIDES

**FIG 3:**
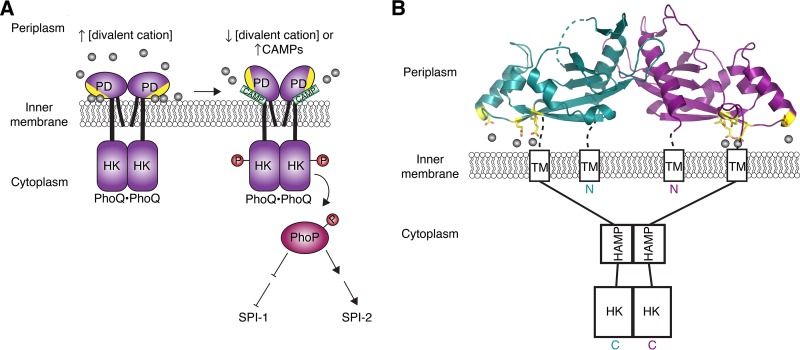
SP1-1 and SPI-2 T3SS regulation by CAMPs in *Salmonella*. (A) CAMPs are sensed by the PhoQ HK. When CAMPs are not present, divalent cations (gray spheres) bridge the acidic patch (yellow) of the PhoQ PD and the negatively charged IM phosphate groups, tethering the PhoQ PD to the membrane and locking it in a repressed state. When CAMPs are present, they compete for binding to the PhoQ PD acidic patch and displace the bound divalent cations, which frees the PhoQ PD from its membrane-locked state and induces a conformational change that activates PhoQ HK activity. The activated HK domain of PhoQ then phosphorylates the RR PhoP, which in turn induces SPI-2 expression and represses SPI-1 expression. (B) The crystal structure of the *S*. Typhimurium PhoQ PD dimer in a divalent cation-bound (gray spheres) state (PDB ID 1YAX). The N- and C-terminal TM helices, HAMP domain, and HK domain are represented as rectangles. Acidic patch residues (yellow sticks) coordinate cations (gray spheres) at the IM outer leaflet interface.

Cationic antimicrobial peptides (CAMPs) are produced by the host as part of the immune response to bacterial, viral, protozoan, or fungal infections (47). Due to their net positive charge and amphipathicity, CAMPs can interact with and permeate phospholipid membranes. Many bacterial pathogens, such as *Salmonella enterica* serovar Typhimurium (48), several streptococcal species (49), *Neisseria meningitidis* (50), and *Pseudomonas aeruginosa* (51), have evolved mechanisms of CAMP resistance. *S*. Typhimurium achieves this by sensing the CAMPs and modifying the lipopolysaccharides (LPSs) that decorate its cell surface. In *S*. Typhimurium, the genes involved in LPS modification are regulated by the PhoPQ two-component system (TCS) (48, 52). This TCS is composed of PhoQ, a membrane-spanning HK, and PhoP, a DNA-binding RR. In addition, the PhoPQ TCS also controls the expression of *Salmonella* pathogenicity islands 1 (SPI-1) and SPI-2, each of which encodes a T3SS (Fig. 3A) (53). PhoQ can function as both an HK and a phosphatase and thereby modulate the phosphorylation status of PhoP in response to environmental cues, such as CAMPs, divalent cations, and acidic pH (54, 55). Phosphorylated PhoP induces expression of SPI-2 T3SS genes and represses the expression of most SPI-1 T3SS genes (Fig. 3A) (56, 57). The SPI-1 T3SS is used for the initial invasion of nonphagocytic cells, and the SPI-2 T3SS is essential for maintaining the *Salmonella*-containing vacuole (SCV) and survival within phagocytic cells (58–60).

Crystallographic studies have demonstrated that divalent cations bind to an acidic patch created by the PhoQ-DcuS-CitA (PDC) sensor fold within the periplasmic domain (PD) (Fig. 3B) (61). *In vitro* studies using recombinant truncations of PhoQ reconstituted in lipid vesicles have shown that PhoQ can directly sense CAMPs through its PD (55). Binding of divalent cations to this acidic patch represses PhoQ HK activity (61, 62). The acidic patch is also important for CAMP recognition, suggesting that CAMPs and divalent cations compete for binding to this region (55). *In vivo*, divalent cations are thought to be coordinated between the PhoQ PD and the phosphate groups of the outer leaflet of the inner membrane (IM) (Fig. 3A and B) (61). The divalent cations bridge the negative charges of the PD acidic patch and the IM phosphates, thus tethering the PhoQ PD to the membrane. Based on nuclear magnetic resonance studies, the metal-bound, and therefore membrane-tethered, PhoQ PD is believed to be limited in structural flexibility and locked in a state that inactivates the HK domain (Fig. 3A) (55). However, when *Salmonella* is exposed to host CAMPs, the positively charged peptides compete for binding to the PhoQ acidic patch and displace bound divalent cations. This displacement is thought to release the PhoQ sensor domain from its membrane-locked state and induce a conformational change that promotes PhoQ HK activity (Fig. 3A) (55, 61).

The exact nature of the conformational change induced by CAMPs, as well as the mechanism of PhoQ HK activation, remains unclear. In a recent study, however, multistate Bayesian modeling of disulfide cross-linking data and existing structures of PhoQ homologous domains predicted that PhoQ transitions between two major states. These states are defined by the insertion or displacement of the PD acidic patch in the IM (Fig. 3A) (63). The authors found that insertion or removal of the PD acidic patch within the membrane was coupled with scissoring transitions, or diagonally opposing displacements, in the PD sensor domain, transmembrane (TM) helical bundles, HAMP, and DHp domains. These changes in conformation are predicted to alter HK domain activity (Fig. 3A and B), but further studies are needed to confirm these changes and to elucidate how they mechanistically alter PhoQ’s HK activity.

## FATTY ACIDS

Fatty acids (FAs) in the intestinal tract are generated through the combined action of the host metabolism and the gut flora. Long-chain fatty acids (LCFAs) enter the small intestine through the diet and via fat metabolism (64). The resident microbiota in the large intestine produces abundant quantities of short-chain fatty acids (SCFAs), including acetate, propionate, and butyrate (reviewed in reference 65). Thus, host and bacterial metabolism result in different LCFA and SCFA concentrations along the GI tract. In addition to their metabolic roles, LCFAs and SCFAs act as cues for bacteria to sense their location in the GI tract and modulate the expression of their virulence factors.

**FIG 4:**
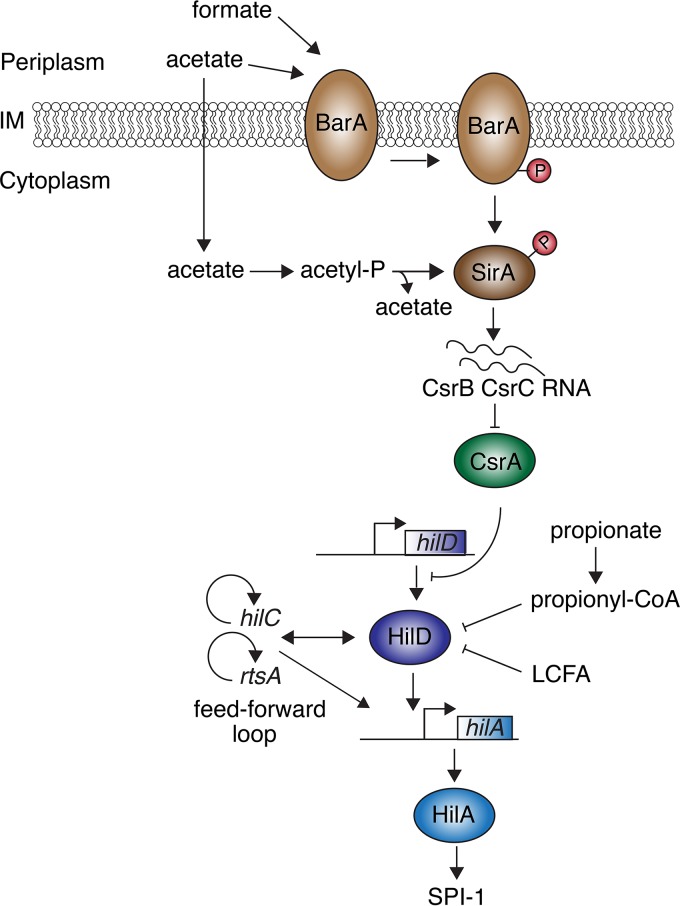
Long- and short-chain fatty acids regulate SPI-1 expression in *Salmonella*. The SCFAs acetate and formate activate BarA HK activity and phosphotransfer to the SirA RR. The acetate metabolite, acetyl-P, can also directly phosphorylate SirA independently of BarA HK activity. P-SirA induces expression of CsrB and CsrC RNAs, which sequester the CsrA protein, thereby alleviating CsrA-mediated repression of *hilD* translation. The HilD protein then induces expression of the regulators HilC and RtsA, which through a feed-forward loop further amplify HilD expression. HilD, HilC, and RtsA also activate expression of HilA, which in turn induces expression of SPI-1 T3SS genes. Conversely, both LCFAs and proprionate negatively repress HilD activity. LCFAs bind to HilD and reduce its affinity for target DNA, including the *hilA* promoter. The propionate metabolite propionyl-CoA destabilizes the HilD protein, thereby inhibiting its transcription factor activity and activation of SPI-1 expression.

Individual FAs have differential effects on *S*. Typhimurium virulence. For example, acetate induces invasion genes, while propionate, butyrate, and LCFAs reduce invasiveness (66–68). Regulation of *S*. Typhimurium virulence by FAs is dependent on the multilayered regulatory network that controls the expression of the invasive SPI-1 T3SS (Fig. 4). The BarA/SirA TCS, which is composed of the hybrid HK BarA and the DNA-binding RR SirA (also known as UvrY), is at the top of this network. The BarA/SirA TCS is activated by various SCFAs through different mechanisms (Fig. 4). Acetate activates SirA via the metabolite acetyl-phosphate, which phosphorylates SirA directly (66). This sensing mechanism bypasses BarA, as it depends on the conversion of acetate to acetyl-phosphate by metabolic enzymes (66). While the role of BarA in SCFA sensing for *S*. Typhimurium is not clear, work on its homologue in *E. coli* demonstrated that BarA senses formate (69), a signal that induces SPI-1 in *S*. Typhimurium (68). In addition, deletion of acetyl-phosphate synthesis genes in *E. coli* still results in low-level activation of a SirA-dependent operon in a BarA-dependent manner when acetate is present, suggesting that BarA also senses acetate (69). Once phosphorylated, the SirA RR activates the expression of CsrB and CsrC, two regulatory RNAs that repress the RNA-binding protein CsrA (Fig. 4) (70, 71). CsrB and CsrC sequester CsrA, preventing it from binding to the *hilD* mRNA Shine-Dalgarno sequence. The free *hilD* mRNA can then be translated.

LCFA and propionate negatively affect SPI-1 T3SS expression by modulating HilD activity via different mechanisms (Fig. 4) (67, 72). LCFAs bind to HilD and reduce its affinity for DNA, thus reducing transcription from HilD-dependent promoters (72). HilD controls its own expression, as well as that of the regulators *hilC* and *rtsA*, resulting in a feed-forward loop that further amplifies LCFA suppression of SPI-1 expression (Fig. 4) (3). The effect of propionate is less direct, as its metabolite, propionyl coenzyme A (CoA), destabilizes HilD and thereby limits its activity (Fig. 4) (67). Golubeva et al. (72) proposed that absorption along the small intestine results in a low concentration of LCFAs in the distal ileum, which would relieve HilD repression and allow the expression of the SPI-1 activator *hilA* at this site. Since the propionate concentration is high in the colon and cecum, this molecule has been proposed to serve as a signal for *S*. Typhimurium that it has left the optimal site, the ileum, for infection (67). The repressive effects of certain FAs in *Salmonella* virulence and their antimicrobial activities have resulted in their use as supplements in animal feed (73). In addition to their direct effect on bacterial pathogens, SCFAs also influence host physiology in diverse ways, including alteration of immune function (74). Thus, the effects of SCFAs on gut pathogens such as *Salmonella* are probably due to a combination of their inhibitory effect on virulence, their antimicrobial properties, and the regulation of the host immune response.

## IRON

**FIG 5:**
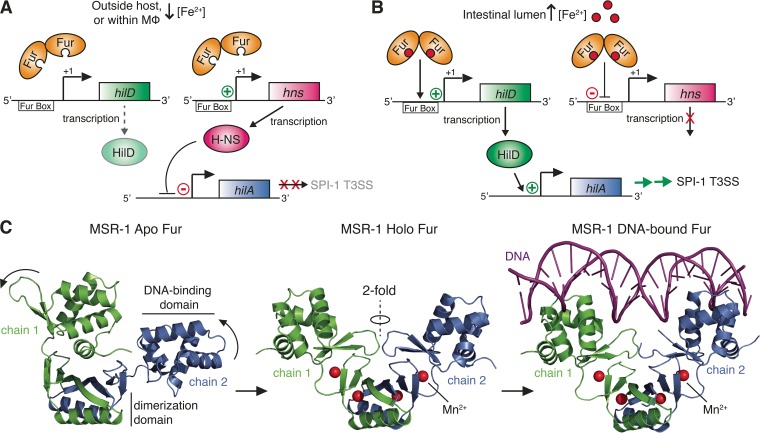
Iron sensing and SPI-1 regulation by Fur in *Salmonella*. (A) Under iron-limiting conditions, apo-Fur cannot bind the Fur boxes upstream of the *hilD* or *hns* promoters and does not activate or repress expression of HilD or H-NS, respectively. H-NS then blocks transcription of *hilA*, the primary transcriptional activator of the SPI-1 T3SS. (B) Under iron-rich conditions (e.g., within the intestinal lumen), Fur binds free iron (Fe^2+^), inducing a conformational change that enables binding to Fur boxes upstream of *hilD* and *hns*. DNA-bound Fur then represses H-NS expression and activates expression of HilD, a transcriptional activator of *hilA*, resulting in HilA expression and downstream SPI-1 T3SS expression. (C) Crystal structures of *M. gryphiswaldense* MSR-1 apo, holo, and DNA-bound Fur. Binding of divalent cations (Mn^2+^, Fe^2+^) induces hinge-like movement of the DNA-binding domains of each monomer, creating a two-fold rotational axis within the holo-dimer and promoting DNA binding (PDB IDs 4RAY, 4RAZ, and 4RB2).

Along with their eukaryotic hosts, most bacterial species require access to free iron for survival and replication (75, 76). Due to the toxicity of free ferric Fe^3+^ and host iron-sequestering mechanisms, the concentration of free iron is low in most host tissues (76). However, in the lumen of the small intestine, which is the primary site of iron absorption, free iron concentrations are high (77). *S*. Typhimurium uses the iron concentration to sense when it has entered the intestinal lumen (78). Many of the genes that are repressed by high Fe^2+^ concentrations are also repressed when *S*. Typhimurium is confined to the intestinal lumen (79). Iron concentration has been found to directly affect the expression of the *S*. Typhimurium SPI-1 T3SS. The activation of SPI-1 expression in response to high iron concentrations depends on the ferric uptake regulator Fur (78). Both metal chelation by 2,2-dipryridyl and deletion of *fur* have been shown to greatly reduce the transcription of the SPI-1 master regulator *hilA* under otherwise-SPI-1-inducing conditions (78). One mechanism by which Fur modulates *hilA* is by repressing expression of the gene encoding the global regulator H-NS (80). Under iron-limiting conditions, such as outside the host or within host macrophages, H-NS directly binds and represses transcription from the *hilA* promoter (Fig. 5A). Under iron-replete conditions, such as in the intestinal lumen, iron-bound Fur (Fur ⋅ Fe^2+^) binds to a regulatory site, termed the Fur box, in the *hns* promoter region and represses *hns* expression. By this mechanism, Fur ⋅ Fe^2+^ indirectly activates HilA and downstream SPI-1 expression (Fig. 5B) (81). In addition to acting through H-NS, Fur ⋅ Fe^2+^ also directly activates expression of *hilD*, a gene encoding the major transcriptional activator for *hilA* (82). Fur ⋅ Fe^2+^ binds to a Fur box upstream of the *hilD* promoter (positions −191 to −163) and activates the expression of *hilD*, which in turn activates the expression of *hilA* (Fig. 5B) (82). In most bacterial species, Fur ⋅ Fe^2+^ binds in the −35 to +12 region of target promoters and represses transcription through steric hindrance (83). However, in *Salmonella*, Fur ⋅ Fe^2+^ binds farther upstream in the *hilD* promoter region, thereby possibly activating *hilD* expression by directly recruiting RNA polymerase (RNAP) or by altering the conformation of the promoter DNA to facilitate RNAP loading (82).

Fur is a major regulator of gene expression that is conserved in several bacterial species. It controls the expression of iron homeostasis genes as well as diverse iron-sensitive cellular processes, including respiration, DNA synthesis, and redox stress resistance (84, 85). In *E. coli* as well as many other bacterial species, Fur binds the Fur box in an Fe^2+^-dependent manner (86). The structural basis for the activation of Fur as a transcription factor by metal ions has not been specifically studied in *S*. Typhimurium, but structural studies performed on Fur homologues have begun to elucidate this mechanism. The Fur domain structure consists of an N-terminal DNA-binding domain (DBD) and a C-terminal dimerization domain linked by a disordered hinge region (Fig. 5C). A recent study of *Magnetospirillum gryphiswaldense* MSR-1 Fur, which solved high-resolution structures of apo, holo, and operator-bound forms of MSR-1 Fur, demonstrated that metal ion binding induces a caliper-like rotation and movement of the DBDs, orienting them for efficient DNA binding and stabilizing the structure (Fig. 5C) (87). Since no completely apo structures of any additional Fur homologues have been published, it is not known if a similar conformational change is induced by metal ion binding in Fur proteins from other species. However, all published holo structures of Fur (i.e., for *E. coli*, *P. aeruginosa*, and *V. cholerae*) adopt the same V-shaped conformation with the dimerization domains at the nexus and DNA-binding domains at the periphery (88).

## BILE SALTS

**FIG 6:**
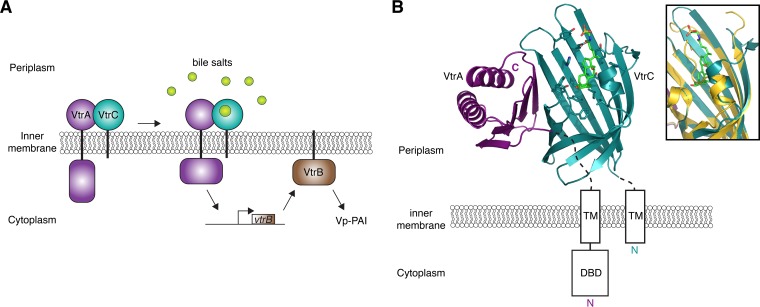
Bile salt sensing and regulation of the Vp-PAI by VtrABC. The *V. parahaemolyticus* IM proteins VtrA and VtrC form a complex through interactions between their PDs. As *V. parahaemolyticus* enters the small intestine, bile salts bind to the VtrA-VtrC complex and activate the DBD of VtrA to induce *vtrB* transcription. VtrB then directly activates the transcription of T3SS2 and other Vp-PAI genes. (B) The crystal structure of the VtrA (purple) and VtrC (teal) PD complex bound to the bile salt taurodeoxycholate (green sticks) (PDB ID 5KEW). TM helices of both VtrA and VtrC are represented as rectangles, as is the DBD of VtrA. An overlay of the bile salt-bound (teal) and apo (yellow) structures illustrates the conformational change that results from bile salt binding (inset).

Bile salts are a component of biliary secretions that are released into the small intestine to solubilize fats. They also have antimicrobial properties against bacteria that inhabit or transit the gastrointestinal tract (89). Successful enteric pathogens have developed ways to tolerate bile salt-induced stress and to use these compounds as markers of intestinal location. The marine bacterium *Vibrio parahaemolyticus* infects humans through the consumption of contaminated raw or undercooked seafood, causing acute gastroenteritis. *V. parahaemolyticus* enterotoxicity is largely mediated by a pathogenicity island (Vp-PAI) which encodes T3SS2, one of two T3SSs in this bacterium. T3SS2 expression is induced by bile salts via signaling, which includes the IM proteins VtrA, VtrB, and VtrC (Fig. 6A) (90–92). VtrA and VtrB are transcription factors that are embedded in the IM by a single transmembrane α-helix (Fig. 6A). VtrA also contains a C-terminal periplasmic domain that interacts with the periplasmic domain of VtrC, which is also anchored to the inner membrane. VtrA and VtrC form a 1:1 complex in which eight β-strands from VtrC and a single β-strand from VtrA form a β-barrel (Fig. 6B). Binding of bile salts to the hydrophobic interior of the VtrA/C dimer displaces a loop that covers the β-barrel in the apo structure (Fig. 6B, inset). This heterodimeric receptor transmits a signal upon bile salt binding that activates the DNA-binding domain of VtrA to induce transcription from the *vtrB* promoter (Fig. 6A) (92). VtrB then promotes transcription of T3SS2-related genes (90, 91). Interestingly, the topologies of the VtrA and VtrB transcription factors tether them to the IM, which may serve to localize T3SS gene products to the membrane site, where they will be assembled and exported. The *V. parahaemolyticus* bile-sensing system is conserved in *V. cholerae* strains that contain a pathogenicity island similar to Vp-PAI. In *V. cholerae* AM-19226, which lacks toxin-coregulated pilus and cholera toxin genes but contains a Vp-PAI-like island, T3SS gene expression depends on the VtrA and VtrB homologues VttR_A_ and VttR_B_ (93). The encoded VtrC homologue is also likely to be part of this regulatory mechanism (93–95).

The inner membrane proteins ToxR and ToxS also play roles in the regulation of T3SS2 expression in *V. parahaemolyticus*. ToxR and ToxS have been linked to bile salt sensing in *V. cholerae*; however, the sensing mechanism remains unclear (96, 97). ToxR has the same domain arrangement as VtrA, with a cytoplasmic DBD, a transmembrane helix, and a periplasmic domain (Fig. 6B). Like VtrC, ToxS is composed of a transmembrane helix and a periplasmic domain. Despite having similar architectures, the periplasmic domains of *V. parahaemolyticus* ToxR and ToxS have less than 25% sequence similarity to VtrA and VtrC, respectively. Recent work identified ToxR in a genetic screen for factors contributing to colonization of the mammalian intestine and showed that ToxR is necessary for *vtrB* expression in *V. parahaemolyticus* (98). This suggests that ToxR works with VtrA and VtrC to regulate T3SS2 expression via VtrB. Work with *V. cholerae* AM-19226 supported a similar role for ToxR in the regulation of this strain’s T3SS (99). ToxR and ToxS also interact through their periplasmic domains (100). Furthermore, Miggett et al. (100) demonstrated that bile salts destabilize the ToxR periplasmic domain, which promotes its interaction with ToxS (100). Additional studies are needed to determine if bile salts have any direct effect on ToxR’s partner, ToxS.

## CONCLUSIONS

**FIG 7:**
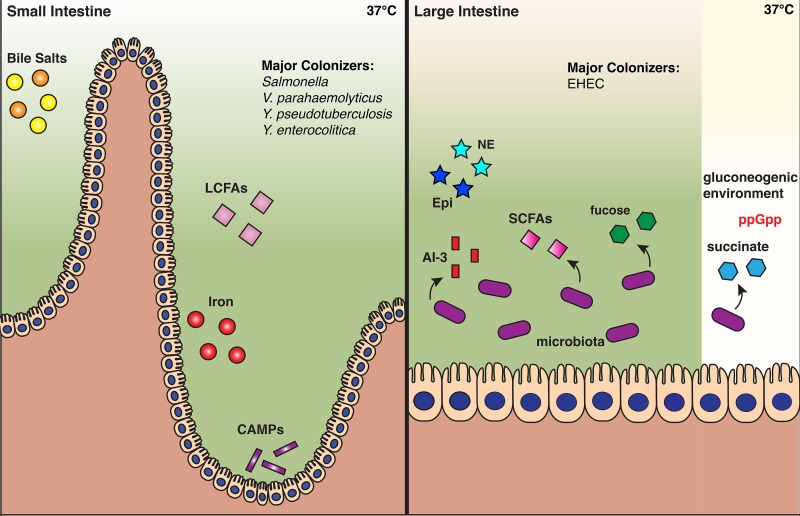
Signals that regulate T3S in the mammalian GI tract. Spatial depiction of the signals that govern T3S in the small and large intestine. Certain signals, like bile salts and iron, are known to be constrained to a specific compartment, based on mammalian physiology, while others are depicted in the compartment where they are postulated to be at the greatest concentration and play the largest role in T3S regulation. Pathogens are listed based on their known major site of colonization within mammalian hosts. Although many host signals and enteric pathogens exist, this figure illustrates only the signals and specific pathogens discussed in this review.

T3SSs are essential to many bacterial pathogens for successful host colonization and invasion. Because their production can reduce bacterial growth rates under certain conditions (101) and can trigger the host’s immune response (102), the timing of their expression must be tightly controlled. It is not surprising, then, that T3SS regulatory pathways are often multileveled and complex. Enteric pathogens have devised strategies to sense signals specifically related to the mammalian gut, and these strategies include molecules involved in host nutrition (iron, fatty acids), metabolism (bile salts), homeostasis (hormones), and microbiota by-products (fucose, succinate). These sensing mechanisms have been integrated into the regulatory networks controlling virulence and colonization factors, which often include T3SSs. As seen in the examples presented here, multiple signals often feed into a single regulatory cascade which has evolved complex feedback mechanisms that allow the bacterium to fine-tune T3SS expression in the signal-rich environment of the mammalian GI tract (Fig. 7).

Bacteria use T3SSs in different ways to promote their survival and proliferation within a host. Pathogens that use T3SSs to maintain their extracellular life cycle, like EHEC, inject effectors essential for establishing a strong attachment to intestinal epithelial cells (13). Other pathogens (e.g., *S. flexneri* and *V. parahaemolyticus*) use their T3SSs both extracellularly, to promote attachment and invasion, and intracellularly, to enhance their proliferation, facilitate vacuolar escape, and mediate cell-to-cell spread (103–106). *Salmonella* has two T3SSs that are used at different stages of infection and are often oppositely regulated by related signals to accommodate the complex wiring of downstream transcriptional regulatory networks.

There is still much to be learned about the signals and sensors that control T3SS expression in enteric bacterial pathogens. For example, it has been postulated that contact between a bacterium and its host cell plays a role in T3SS regulation. Recent work with EHEC suggested that the outer membrane lipoprotein NlpE may regulate T3SS expression through a surface-sensing mechanism involving the Cpx TCS (107, 108). However, NlpE is only required for activation of the LEE*-*encoded T3SS in response to attachment to abiotic surfaces and undifferentiated Caco-2 cells (107). It is possible that other lipoproteins, such as YafY, NlpA, Pal, or OsmB, sense adhesion to other cell types. However, further studies are needed to determine if these other lipoproteins are involved in the regulation of the T3SS in response to surface sensing (109). The mechanism by which the sensor domains NlpE and other outer membrane lipoproteins may sense hydrophobic surfaces is poorly understood and awaits further study. We anticipate that future work will further elucidate how biochemical signals from both the host and the environment are sensed by enteric pathogens to regulate T3S and other mechanisms of virulence. Elucidation of these sensing mechanisms could provide new potential therapeutic targets for the improved management of GI tract infections by enteric bacteria.
